# “The Shelves Are Bare”: The Impact of COVID-19 on Families Enrolled in a Pediatric Produce Prescription Program

**DOI:** 10.7759/cureus.31540

**Published:** 2022-11-15

**Authors:** Amy Saxe-Custack, Heather Lofton, Chantel Dawson, Sarah Egan, Mona Hanna-Attisha

**Affiliations:** 1 Pediatrics and Human Development, Michigan State University–Hurley Children’s Hospital Pediatric Public Health Initiative, Michigan State University College of Human Medicine, Flint, USA; 2 Marriage and Family Therapy, The Family Institute at Northwestern University, Chicago, USA; 3 Medical Oncology, Thomas Jefferson University, Philadelphia, USA; 4 Public Health, Michigan State University–Hurley Children’s Hospital Pediatric Public Health Initiative, Michigan State University College of Human Medicine, Flint, USA

**Keywords:** pediatrics, covid-19, food insecurity, food access, fruit and vegetable prescriptions

## Abstract

Objectives

A large pediatric clinic in Flint, Michigan, implemented a produce prescription program for youth to address enduring challenges with food access and food insecurity. Approximately 18 months later, on March 23, 2020, the State of Michigan issued a “stay home, stay safe” executive order in response to the COVID-19 pandemic. This study sought to (1) explore caregiver experiences with access to and utilization of the prescription program during COVID-19; and (2) understand perceived changes in the food environment during the “stay home, stay safe” executive order.

Methods

Researchers collected data through recorded, semi-structured telephone interviews with caregivers of children who received at least one produce prescription and had previously enrolled in a preliminary effectiveness study on the prescription program. We transcribed the recordings verbatim for textual analysis. Examining the qualitative data using thematic analysis, we identified patterns across transcripts and formulated illustrative themes.

Results

Fifty-six caregivers (mean age, 41.3 ± 10.3 years) participated in interviews. The majority were female (91%), African American (70%), and Flint residents (75%). Recurrent themes, each centered around changes in the food environment resulting from COVID-19, emerged: (1) produce prescription access and utilization; (2) food access constraints; (3) food shopping adjustments; and (4) food insecurity stress. Perceived consequences of COVID-19 included increased anxiety related to food shopping and food insecurity alongside challenges accessing and utilizing the produce prescription program.

Conclusions

This study highlights the many ramifications of the COVID-19 pandemic on vulnerable families. More comprehensive efforts are necessary to address substantial barriers to healthy food access and affordability caused by the recent pandemic.

## Introduction

About 6.5 million US children experience food insecurity and its consequences, including poor diet quality [1-3], negative health and behavioral outcomes [4,5], and low academic achievement [5,6]. With a dramatic rise in unemployment alongside widespread school closures that resulted from the COVID-19 pandemic, food insecurity climbed sharply, placing even more children at risk [7,8]. Efforts to address both food access and food insecurity among youth, even prior to COVID-19, included introducing pediatric produce prescription programs [9-12]. While such programs have various designs and approaches, most entail physicians issuing prescriptions that are used to acquire fresh fruits and vegetables from local farmers’ markets, mobile markets, or food stores. Preliminary research, including a related qualitative study [9], has reported that exposure to pediatric produce prescriptions is associated with favorable changes in food security [12], food shopping [13], and children’s dietary behaviors [9,11,14].

In August 2018, researchers partnered with a large pediatric clinic in Flint, Michigan, to provide one $15 prescription for fresh produce to every patient during office visits [10]. The downtown farmers’ market and a local mobile market were participating vendors. On March 23, 2020, approximately 18 months after the produce prescription program’s introduction, the State of Michigan issued a “stay home, stay safe” executive order in response to the COVID-19 pandemic. The order resulted in the immediate closure of schools, early childcare facilities, and non-essential businesses. Furthermore, visits to pediatric offices in Flint, and across the country, plummeted [15]. With the shift from in-person clinic visits to telehealth appointments, prescriptions were distributed virtually via an online patient medical portal. The downtown farmers’ market closed at the beginning of the pandemic; however, the mobile market continued to provide contactless home delivery of produce boxes.

This study is a qualitative exploration with caregivers of children who participated in the produce prescription program. We sought to: (1) explore caregiver experiences with access to and utilization of the prescription program during COVID-19; and (2) understand perceived changes in the food environment during the “stay home, stay safe” executive order. This article was previously presented as a poster abstract at the 2022 Society for Nutrition Education and Behavior (SNEB) Annual Conference on July 30, 2022 [16].

## Materials and methods

Study setting and population

In Flint, Michigan, the child poverty rate is nearly 60% [17], and the community continues to battle a lead-in-water public health crisis [18] that is worsened by poor nutrition [19]. Flint, like many low-income cities, has insufficient resources and dietary options. Grocery stores are limited in Flint, and local stores are more likely to carry lower-quality foods and fewer healthy options than those in higher-income neighborhoods [9,20-22].

In August 2018, a pediatric office in Flint with approximately 3,000 active patients, most of whom receive public health insurance, introduced a produce prescription program. The program provided all patients (0-18 years of age) with one $15 prescription, redeemable at the downtown farmers’ market or local mobile market, during each office visit. The mobile market also offered fresh produce boxes with free delivery that could be ordered via telephone or website. Vendors treated prescriptions as gift certificates that were redeemable solely for fresh fruits and vegetables.

Between August 2018 and March 2019, 365 caregiver-child dyads provided written consent/assent to participate in a study to assess the feasibility and preliminary effectiveness of the produce prescription program [10], including follow-up interviews one year after receiving their first $15 prescription. All participants spoke English.

Approach and theoretical framework

This study’s design and approach were based on the theoretical framework of Bandura’s Social Cognitive Theory, which explains behavior through a dynamic, three-stage model that links personal factors, environmental factors, and behavior [23]. Because caregivers generally guide children in their dietary intake [24,25], a qualitative exploration of environmental factors that facilitate or impede easy access to fresh, high-nutrient foods was particularly important. Therefore, in collaboration with local pediatricians and agricultural leaders, the framework of Social Cognitive Theory in this study supported a sub-focus on environmental changes that occurred during the pandemic.

Data collection

We collected data through semi-structured telephone interviews between April 2020 and June 2020. All caregivers who enrolled in the original study between August 2018 and March 2019 were eligible to participate. To assess experiences with the prescription program during COVID-19 as well as caregiver perceptions of the food environment during the executive order, we developed an open-ended interview process. The original interview guide was based on existing literature, research questions, and our experiences with the topic and population.

Before initiating the study, we completed four pilot interviews with caregivers. Following these, we jointly decided to add one question to the original interview guide that was specific to food access challenges during the executive order. After finalizing the interview guide (Table 1), two researchers independently facilitated 56 additional telephone interviews. Participants did not receive compensation.

**Table 1:** Interview Guide to Explore Caregiver Experiences with Pediatric Produce Prescription Program During COVID-19 and Perceptions of Food Environment During Stay-Home Executive Order (April 2020–June 2020)

Questions/Prompts
1. Tell me about your experiences with the fruit and vegetable prescription program.
2. How (if at all) has participation in the fruit and vegetable prescription program changed since the “stay home, stay safe” executive order was issued as a result of the coronavirus (COVID-19) pandemic (March 23^rd^)?
3. How (if at all) has your source of food/groceries changed since the “stay home, stay safe” executive order was issued as a result of the coronavirus (COVID-19) pandemic (March 23^rd^)?
4. How (if at all) has COVID-19 and the “stay home, stay safe” executive order impacted your ability to access food?
5. What kinds of nutrition programs would be most useful to you and your family?
6. Is there anything else that you would like to share about your experiences?

Analysis

We audio recorded all telephone interviews and transcribed the recordings verbatim for textual analysis. While examining data with a multi-step coding process guided by thematic analysis, we uncovered and analyzed patterns across the transcripts, which ultimately formed illustrative themes [26]. During the initial coding process, three researchers individually reviewed and labeled preliminary meaningful patterns for thematic purposes. We then collapsed similar themes to confirm the final emerging themes. Lastly, we selected explanatory direct quotations to support the final themes and sub-themes.

We received institutional review board approval from Michigan State University (approval number: STUDY00000666).

## Results

Among the 56 interview participants (mean age, 41.3 ± 10.3 years), the majority were female (91%), African American (70%), and Flint residents (75%) (Table 2). Characteristics of interview participants closely matched those of the entire sample of 365 caregivers who enrolled in the larger study (mean age 39.7 ± 9.9 years), with most female (91%), African American (66%), and Flint residents (72%). After these 56 subjects completed telephone interviews, researchers noted no new information was forthcoming; we had reached data saturation. We present the findings of the following recurrent themes that emerged, each centered around changes in the food environment that resulted from COVID-19 and the subsequent executive order: 1) produce prescription access and utilization; 2) food access constraints; 3) food shopping adjustments; and 4) food insecurity stress. These themes are organized by associated sub-themes in Table 3.

**Table 2:** Characteristics of the Study Sample (April 2020–June 2020) Abbreviations: SD, standard deviation; y, years

Caregiver Characteristic	n	% or Mean (SD)
Gender		
Male	5	9%
Female	51	91%
Age, y		
Caregiver	56	41.30 (10.31)
Ethnicity		
African American	39	70%
White	12	21%
Other	5	9%
Residence		
Flint	42	75%
Non-Flint	14	25%
Education		
Some high school/High school	19	34%
Trade school/Some college no degree	4	7%
Associate degree	21	38%
Bachelor’s degree	3	5%
Graduate or professional degree	9	16%

**Table 3:** Representative Quotes Collected from Caregivers of Children Enrolled in a Produce Prescription Program (April 2020–June 2020)

Theme	Sub-theme	Illustrative Quote
1. Prescription Program Access and Utilization	1.1 Fear of Exposure	It’s the stress of just being out there in the unknown and going out and coming home. Trying to take a shower and not giving anything to my kids because they haven’t been anywhere. Trying to keep the virus out of my house. (ID 326, African American Female, Age 40)
		I just can’t wait until this is all over with, so I can go to the doctor. And my daughter, her tooth aches. I think she has a real bad cavity. (ID 041, African American Female, Age 26)
	1.2 Shift to Mobile Market Produce	As far as going to the farmers’ market, we haven’t been down there. But we order the produce, and they bring it to us… they have it packed in a little box all nice and neat. It was some very good-looking items. (ID 280, African American Female, Age 53)
		So, we started using Flint Fresh. They have a wide variety of items, and they deliver as well. They (kids) can go in and make their own selection as to what they want. When they get it, it comes in their own box. They like that it comes in their own box. It’s almost like a present for them. (ID 320, African American Female, Age 41)
		I prefer the delivery one. That one, it was good. It always has great quality…. When we’re making dinner, we make a list of stuff that we will need, and they normally work pretty good around that. We will call and request it, what we need. They work with us really well. (ID 355, White Female, Age 74)
2. Food Access Constraints	2.1 Cost	The budget is low, expenses are high…. I just buy less. I don’t too much switch out stuff. I just buy less. If it gets to where I can’t get certain items, I just skip over them for the time. (ID 358, African American Female, Age 51)
		With the prices and stuff, the last time my daughter went to shop, she went down to Pontiac to some store that had a bunch of deals ’cause the prices have gone up so much. She went all the way to Pontiac for foods that are affordable. (ID 066, White Female, 51)
		I just left the grocery store to get something today, and a pack of hamburger was like $9 to feed 4 people. I guess it’s the time, the prices going up, but it wasn’t a big pack of meat. (ID 345, African American Female, Age 51)
	2.2 Food Shortages and Restrictions	They are selling out. The groceries are selling out pretty fast. It makes it kind of hard for me to get up there and get them. Once you get out there, it’s so cram-packed with people trying to get what they need, you don’t have much left on the fruit and vegetable aisle. (ID 257, African American Female, Age 40)
		[Stores] put a limit on the things you can buy. So, I can’t shop like I normally would. (ID 101, African American Female, Age 32)
		With me feeding so many people, I have to go to the store two or three times in a day to get enough [food] for everybody. (ID 217, White Female, Age 65)
	2.3 Food Quality	There were definitely times when the fruit and vegetable selection was mushed and everything. So, we just skipped it during that shopping trip. And since the virus, that has happened more. (ID 216, White Female, Age 37)
		[The stores] have vegetables pretty much, but the ones that’s left over are like the stuff that doesn’t look so good. (ID 269, African American Female, Age 34)
3. Food Shopping Adjustments	3.1 Modified Shopping Hours	I don’t like being in there when there are a lot of people in the store.... I’ve been going really early in the morning. I work third shift. So, when I get off work at six in the morning, I just wait around until like seven. Then, I go in the stores and it’s not as many people. (ID 317, African American Female, Age 33)
		Now you have to try and beat the crowd, and you have to wake up super early in the morning and try and see if you can get there so you can meet the truck when it arrives. It’s just different.... I try to see if I can get to the store, you know, moderately stocked shelves. (ID 339, African American Female, Age 48)
	3.2 Varied Store Visits	I have to run to a hundred different stores to find things. (ID 306, White Female, Age 31)
		It’s hard to find different things. We have to go from store to store to find certain things that we eat or need for a recipe. (ID 223, African American Female, Age 39)
	3.3 Produce Modifications	Instead of the fresh fruits, we just did the canned peaches or canned pears. (ID 216, White Female, Age 37)
		I get canned fruits sometimes now if the produce is not available. (ID 339, African American Female, Age 48)
		Fresh fruit or vegetables, they don’t last… So, to keep them, we’re trying to do more canned. Trying to get them in light syrup, stuff that’s still healthy. (ID 198, White Female, Age 29)
4. Food Insecurity Stress	4.1 Adequate Food	With the kids, we are used to them in school. So, food lasts a little longer. It doesn’t last as long as it used to. (ID 263, African American Male, Age 31)
		Of course, I am going to worry because they (children) just assume that whenever they come into the kitchen, there is going to be food. But as a parent, you’re stressing out and wondering, “Well, is it going to be enough to last? Will I be able to make it last?” (ID 186, African American Female, Age 62)
		Ain’t nobody going to starve. They may be hungry because they can’t get what they want. But they ain’t going to starve. That’s all that matters to me. (ID 062, African American Male, Age 35)
	4.2 Local Food Assistance	The schools drop off food, but the kids don’t eat it. I feel badly. They pick through it and throw the rest away. (ID 288, White Female, Age 38)
		They are offering something through the school, but I don’t have a vehicle at the moment. (ID 261, Other Race Female, Age 37)

Produce prescription access and utilization

Fear of exposure to COVID-19 created notable challenges related to produce prescription access (Table 3, sub-theme 1.1). Many caregivers discussed how they remained secluded inside their homes to keep children safe from the virus. Some specifically described challenges in receiving produce prescriptions as they deliberately avoided in-person pediatric office visits during the pandemic and, as a result, received fewer prescriptions.

*I don’t go anywhere. My kids actually haven’t been outside because I am afraid to expose them.* (ID 036, Other Race Female, Age 39)

*Giving us free fruits and vegetables, it definitely helps me keep her (daughter) healthier…. It’s just that we can’t go to the doctor to get the (produce) prescriptions. Their last appointment, they just kind of did it over the phone.* (ID 247, African American Female, Age 27)

Before COVID-19, most produce prescriptions were redeemed at the local farmers’ market. Although families continued using produce prescriptions during the pandemic, many shared that prescription purchases shifted away from farmers’ market produce stands to the mobile market, which offered free home delivery of produce boxes. Many caregivers noted that children remained engaged in food shopping by actively selecting fruits and vegetables to fill their produce boxes from the mobile market website (Table 1, sub-theme 1.2).

*[Children] really took ownership of the prescriptions. They both took it upon themselves to go online to shop [at the mobile market] and pick out what fruits and veggies they wanted. I let them pick out what they wanted to eat or what they wanted to try.* (ID 255, African American Female, Age 46)

Food access constraints

Rising food costs during the “stay home, stay safe” executive order influenced most caregivers’ food purchasing behaviors (Table 3, sub-theme 2.1). Caregivers managed these increasing costs in various ways, including by purchasing less food, limiting new or unfamiliar foods, or traveling far distances for sales or wholesale prices.

*I hate it. I hate being wasteful. Food costs too much, and it’s going up. So, that’s what causes people to stick to what they know. It’s so costly, it makes you hesitant to venture out and try new things because if your family doesn’t like it, well, now I’m sitting here with all this wasted money.* (ID 223, African American Female, Age 39)

Caregivers also discussed COVID-related food shortages and purchasing restrictions (Table 3, sub-theme 2.2). Although many referenced a limited supply of specific foods, such as fruits and vegetables, some generally described stores with empty shelves. As a result, most felt food choices were limited, forcing caregivers to purchase whatever foods were available. Some were further challenged by stores that imposed limits on the purchase of various food items.

*They didn’t have no meat. The vegetables was limited. They didn’t even have cabbage. The greens, they didn’t have those. And there’s a limit of two cans on the vegetables, like the corn and the green beans.* (ID 241, African American Female, Age 46)

*You walk into the store, and it looks like a ghost town. The shelves are bare. You have to grab what’s there, and sometimes it may not be what you want. You don’t have a choice. You have to take what you can.* (ID 339, African American Female, Age 48)

Before COVID-19, challenges with food quality were apparent throughout Flint [9,20,22]. Many caregivers believed this worsened during the pandemic, creating additional barriers to accessing fresh, high-quality fruits and vegetables (Table 3, sub-theme 2.3).

*The thing with the vegetables, I found out that they are frozen. They are freezing all the vegetables and the fruit. So, when you get them home, they are frozen on the inside. The quality is horrible. You make a salad, and you have a soggy salad because the inside of the lettuce is frozen.* (ID 320, African American Female, Age 41)

Food shopping adjustments

The pandemic, and related executive order, caused abrupt changes, including the temporary closure of the farmers’ market and modified grocery store hours. These changes necessitated that caregivers adjust their approach to food shopping. Many specifically shared how they altered the time they were accustomed to food shopping (Table 3, sub-theme 3.1). Most indicated they began shopping late in the evening or very early in the morning.

*Everything is closed down, and grocery stores that are open are so overwhelming to where I have to wait until a certain time to try to go out and get groceries…. Either really, really early, late in the evening, or at night.* (ID 358, African American Female, Age 51)

Caregivers also discussed how farmers’ market closures and food shortages in grocery stores required adjustments to typical food shopping behaviors. Many began visiting unfamiliar stores or shopping at multiple stores to find food items (Table 3, sub-theme 3.2).

*I started shopping at different places ’cause when I can’t find something at one store, I go to another one.* (ID 358, African American Female, Age 51)

*I’m not shopping at the farmers’ market now because I know they are closed. They aren’t open to the public.* (ID 331, African American Female, Age 42)

Caregivers discussed modifications to the type of produce they purchased as a result of the pandemic (Table 3, sub-theme 3.4). Many talked about replacing fresh produce, which had a limited shelf-life and was difficult to find in grocery stores, with canned or frozen varieties.

*We don’t have the fresh options that we used to have during our meals, so we eat more canned now.* (ID 101, African American Female, Age 32)

Food insecurity stress

As the pandemic caused a shift in responsibility to feeding children at home, rather than school, most caregivers expressed feelings of stress or anxiety related to food insecurity, particularly in relation to providing adequate food for their children (Table 3, sub-theme 4.1). Many worried that the food in the house would not last, and some made attempts to ration food. Several caregivers further shared personal accounts related to anxiety surrounding decisions to purchase groceries or pay bills.

*I had to make that choice to spend it all at one time. Do I pay a bill or do I shop for groceries? So, that was something I hadn’t had to experience in a long time... I am watching how much I cook because sometimes I’ll cook just enough. I’m starting to worry about how long food is going to last now and if it’s going to be affordable for us later. *(ID 320, African American Female, Age 41)

Caregivers, all of whom had school-aged children, talked most frequently about food assistance through local schools. Although caregivers expressed appreciation to school systems that offered free meals to children and families, some also noted challenges (Table 3, sub-theme 4.2). Barriers to participation in school lunch and breakfast programs included a lack of variety, small portions, and limited transportation options.

*I used to go to [the children’s school] to get the snacks and lunches for the children. After they give you the same thing for four or five weeks, the kids came to not like it anymore. It’s the same thing over and over and over. So, they are getting burnt out on it, and they are wasting it.* (ID 257, African American Female, Age 40)

## Discussion

Interviews occurred soon after Michigan enacted an executive order, while many remained at home, unable to work and facing the possibility of unemployment. Most caregivers in this study were already challenged with limited access to fresh, high-nutrient foods [10,22] and talked extensively about additional access barriers caused by the pandemic. These additional barriers required that caregivers modify usual food shopping behaviors. Many began shopping at multiple stores or at varied times simply to find the foods they needed. Some caregivers reduced, or even eliminated, fresh food purchases due to limited availability or fear of spoilage. A perceived consequence of COVID-19 was this decline in purchasing of both fresh as well as unfamiliar foods. This finding is notable as previous qualitative work has indicated that pediatric produce prescriptions are an effective tool to persuade children to try new fruits and vegetables [9]. Although growing evidence supports the positive influence of produce prescription programs on child dietary behaviors [9,11,14], our study suggests that this influence may have been counteracted by substantial environmental barriers to healthy food access resulting from COVID-19.

Caregivers reported continued use of produce prescriptions during the pandemic; however, they also discussed challenges with accessing and utilizing prescriptions. Fear of exposure to COVID-19 created a sharp decline in pediatric office visits that led to decreased prescription distribution during the pandemic’s early months. In an effort to support distribution during telehealth visits, the clinic developed and implemented virtual prescriptions that were disseminated by pediatricians during virtual and in-person office visits using the electronic medical record (EMR) system. However, virtual prescriptions were largely underutilized during the pandemic and not a sufficient substitute for the paper prescriptions used prior to the pandemic. Additionally, the temporary closure of the farmers’ market, which served as the primary vendor for produce prescriptions before the pandemic [9], limited prescription redemption to the mobile market during the height of the pandemic. This prompted some families to shop at the mobile market, where children could select their own fruits and vegetables via the mobile market website and exchange their prescriptions for home-delivered produce boxes. The contactless delivery and farm fresh produce offered by the mobile market addressed many challenges voiced by participants during COVID-19, including eliminating the need to visit busy grocery stores and supplying ample, quality produce.

Although caregivers expressed appreciation for a produce prescription program that they viewed as helpful during the pandemic, it is unlikely that the program alone was sufficient to offset the severe, extensive consequences of COVID-19 on food insecurity and hunger. Our study highlights the many ramifications of this pandemic on vulnerable children and families, many of whom were at elevated risk for hunger and food insecurity before the pandemic [1]. In addition to decades-long challenges related to food access and affordability, Flint’s children and families are struggling with the effects of a population-wide lead-in-water public health crisis [18]. Even outside these public health crises, Flint faces extraordinary health challenges, with many families and individuals living in poverty, under- or unemployed. As cities across the country struggle to manage COVID-19 and its devastating effects, there is an urgent need for public health programs to work together to collectively address enduring challenges, such as child poverty and related food insecurity, which exacerbated the negative impacts of this pandemic. 

Pediatricians, and primary care providers, are a leading source of health information [27,28] and should play a key role in both identifying and addressing food insecurity among children and families. This includes not only screening households for food insecurity, but also working collaboratively with community-based organizations to provide resources for families in need. Moreover, efforts should be made to support nutrition security such that foods provided to vulnerable children intentionally support healthy growth and development. Our study highlights challenges with pediatric produce prescriptions during an unprecedented global pandemic that negatively impacted not only this program, but the entire food environment in Flint. Conversely, the mobile market as a prescription vendor provided a safe and contactless fresh produce option that facilitated continued engagement in the program. Although produce prescriptions appear to be an innovative and effective tool to address enduring challenges with food access and food insecurity among low-income children and families [9,11-14], our study suggests that more comprehensive efforts are necessary to tackle substantial COVID-related barriers to healthy food access and affordability.

This study has several limitations to note. As a cross-sectional study, the findings do not offer insights into whether or how participants’ experiences may have varied as the COVID-19 pandemic persisted. There was also the possibility of selection bias as feedback from caregivers who participated in interviews may differ from those who did not; however, the characteristics of interview participants closely matched those of the larger study sample. Additionally, the sample size was small and specific to one clinic. Therefore, findings may not be generalizable to a broader population. Although caregivers could have felt inclined to describe positive experiences in hopes that the prescription program would continue, they were candid about challenges with the program, including farmers’ market closure and limited pediatric visits, and we are confident in the sincerity of their responses.

## Conclusions

COVID-19 has exacerbated and exposed the realities of increasing nutritional disparities among US children. Caregivers in the current study indicated increased anxiety related to food shopping and provision of food for their children, increased food insecurity, and challenges accessing and utilizing fresh produce prescriptions for their children. These findings suggest more comprehensive efforts are necessary to successfully address substantial barriers to healthy food access and affordability caused by the recent pandemic. Future research will investigate whether the challenges presented in this paper were sustained over time.

## References

[1] Landry MJ, van den Berg AE, Asigbee FM, Vandyousefi S, Ghaddar R, Davis JN (2019). Child-report of food insecurity is associated with diet quality in children. Nutrients.

[2] Rodríguez LA, Mundo-Rosas V, Méndez-Gómez-Humarán I, Pérez-Escamilla R, Shamah-Levy T (2017). Dietary quality and household food insecurity among Mexican children and adolescents. Matern Child Nutr.

[3] Eicher-Miller HA, Zhao Y (2018). Evidence for the age-specific relationship of food insecurity and key dietary outcomes among US children and adolescents. Nutr Res Rev.

[4] Casey PH, Simpson PM, Gossett JM (2006). The association of child and household food insecurity with childhood overweight status. Pediatrics.

[5] Shankar P, Chung R, Frank DA (2017). Association of food insecurity with children's behavioral, emotional, and academic outcomes: a systematic review. J Dev Behav Pediatr.

[6] Faught EL, Williams PL, Willows ND, Asbridge M, Veugelers PJ (2017). The association between food insecurity and academic achievement in Canadian school-aged children. Public Health Nutr.

[7] Adams EL, Caccavale LJ, Smith D, Bean MK (2020). Food insecurity, the home food environment, and parent feeding practices in the era of COVID-19. Obesity (Silver Spring).

[8] Kinsey EW, Kinsey D, Rundle AG (2020). COVID-19 and food insecurity: an uneven patchwork of responses. J Urban Health.

[9] Saxe-Custack A, Lofton HC, Hanna-Attisha M, Victor C, Reyes G, Ceja T, LaChance J (2018). Caregiver perceptions of a fruit and vegetable prescription programme for low-income paediatric patients. Public Health Nutr.

[10] Saxe-Custack A, LaChance J, Hanna-Attisha M, Ceja T (2019). Fruit and vegetable prescriptions for pediatric patients living in Flint, Michigan: a cross-sectional study of food security and dietary patterns at baseline. Nutrients.

[11] Ridberg RA, Bell JF, Merritt KE, Harris DM, Young HM, Tancredi DJ (2019). Effect of a fruit and vegetable prescription program on children's fruit and vegetable consumption. Prev Chronic Dis.

[12] Ridberg RA, Bell JF, Merritt KE, Harris DM, Young HM, Tancredi DJ (2019). A pediatric fruit and vegetable prescription program increases food security in low-income households. J Nutr Educ Behav.

[13] Saxe-Custack A, Sadler R, LaChance J, Hanna-Attisha M, Ceja T (2020). Participation in a fruit and vegetable prescription program for pediatric patients is positively associated with farmers' market shopping. Int J Environ Res Public Health.

[14] Saxe-Custack A, LaChance J, Hanna-Attisha M (2019). Child consumption of whole fruit and fruit juice following six months of exposure to a pediatric fruit and vegetable prescription program. Nutrients.

[15] Sulaski Wyckoff A (2021). AAP News: Delayed care: AAP responds to report on drop in pediatric visits in Medicaid, CHIP. https://www.aappublications.org/news/2020/09/28/delayedcare092820.

[16] Saxe-Custack A, Lofton H, Egan S, Dawson C, Hanna-Attisha M (2022). P056 challenges and successes of a pediatric produce prescription program during COVID-19. J Nutr Educ Behav.

[17] U.S. Census Bureau: 2014-2018 American Community (2021). U.S. Census Bureau: 2014-2018 American Community Survey 5-Year Estimates, Table DP03: selected economic characteristics. https://data.census.gov/cedsci/table?q=DP03&g=1600000US2629000&tid=ACSDP5Y2018.DP03&hidePreview=true.

[18] Hanna-Attisha M, LaChance J, Sadler RC, Champney Schnepp A (2016). Elevated blood lead levels in children associated with the Flint drinking water crisis: a spatial analysis of risk and public health response. Am J Public Health.

[19] Ahamed M, Siddiqui MK (2007). Environmental lead toxicity and nutritional factors. Clin Nutr.

[20] Saxe-Custack A, Lofton HC, Hanna-Attisha M, Tata Z, Ceja T, LaChance J (2019). Caregiver experiences with an innovative farmers' market incentive program for children in Flint, Michigan. Glob Pediatr Health.

[21] Shaver ER, Sadler RC, Hill AB (2018). The Flint Food Store Survey: combining spatial analysis with a modified Nutrition Environment Measures Survey in Stores (NEMS-S) to measure the community and consumer nutrition environments. Public Health Nutr.

[22] Mayfield KE, Carolan M, Weatherspoon L, Chung KR, Hoerr SM (2017). African American women's perceptions on access to food and water in Flint, Michigan. J Nutr Educ Behav.

[23] Bandura A (2004). Health promotion by social cognitive means. Health Educ Behav.

[24] Draxten M, Fulkerson JA, Friend S, Flattum CF, Schow R (2014). Parental role modeling of fruits and vegetables at meals and snacks is associated with children's adequate consumption. Appetite.

[25] Haß J, Hartmann M (2018). What determines the fruit and vegetables intake of primary school children? - an analysis of personal and social determinants. Appetite.

[26] Braun V, Clarke V (2012). Thematic analysis. APA Handbook of Research Methods in Psychology, Vol. 2. Research Designs: Quantitative, Qualitative, Neuropsychological, and Biological.

[27] Lupi JL, Haddad MB, Gazmararian JA, Rask KJ (2014). Parental perceptions of family and pediatrician roles in childhood weight management. J Pediatr.

[28] Daniels SR, Hassink SG (2015). The role of the pediatrician in primary prevention of obesity. Pediatrics.

